# Perspectives and Prospective on Solid Lipid Nanoparticles as Drug Delivery Systems

**DOI:** 10.3390/molecules27051543

**Published:** 2022-02-24

**Authors:** Osama A. Madkhali

**Affiliations:** Department of Pharmaceutics, College of Pharmacy, Jazan University, Jazan 45124, Saudi Arabia; omadkhali@jazanu.edu.sa

**Keywords:** solid lipid nanoparticles, drug delivery, antibiotics, anticancer

## Abstract

Combating multiple drug resistance necessitates the delivery of drug molecules at the cellular level. Novel drug delivery formulations have made it possible to improve the therapeutic effects of drugs and have opened up new possibilities for research. Solid lipid nanoparticles (SLNs), a class of colloidal drug carriers made of lipids, have emerged as potentially effective drug delivery systems. The use of SLNs is associated with numerous advantages such as low toxicity, high bioavailability of drugs, versatility in the incorporation of hydrophilic and lipophilic drugs, and the potential for production of large quantities of the carrier systems. The SLNs and nanostructured lipid carriers (NLCs) are the two most frequently used types of nanoparticles. These types of nanoparticles can be adjusted to deliver medications in specific dosages to specific tissues, while minimizing leakage and binding to non-target tissues.

## 1. Introduction

Solid lipid nanoparticles (SLNs) are developed as colloidal carrier systems for delivery of water-soluble drugs and for successful correction of dynamic therapy [1]. A recent report demonstrated that SLNs form a dynamic drug delivery system that is diverse and adaptable. This system has increased potential for improving drug stability and controlling drug release from the matrix over time. Moreover, packaging drugs in SLNs aids in resisting proteolytic degradation before the drugs reach their target sites [2]. A consensus has emerged among researchers that lipids are excellent excipients for drug delivery due to their inherent ability to enhance gastrointestinal solubilization and the absorption of poorly bioavailable drug components by the lymphatic system [3,4]. As alternatives to oil-in-water (O/W) emulsions for parenteral nutrition, SLNs are one of the novel potential colloidal transporter systems used in place of polymers, and they are distinguishable from O/W emulsions. The fluid lipid of the O/W emulsion can be replaced by a solid lipid nanoparticle [5]. Techniques such as high-pressure homogenization and solvent evaporation are used for the production of SLNs [6,7]. Furthermore, the features of SLNs can be changed to improve their efficiency. This is particularly useful for medications with low water solubility. There is need for a thorough literature review on the many types of SLNs and traditional matrices used as drug delivery methods in order to comprehend SLNs and their potential applications in the pharmaceutical industry.

**Figure 1:**
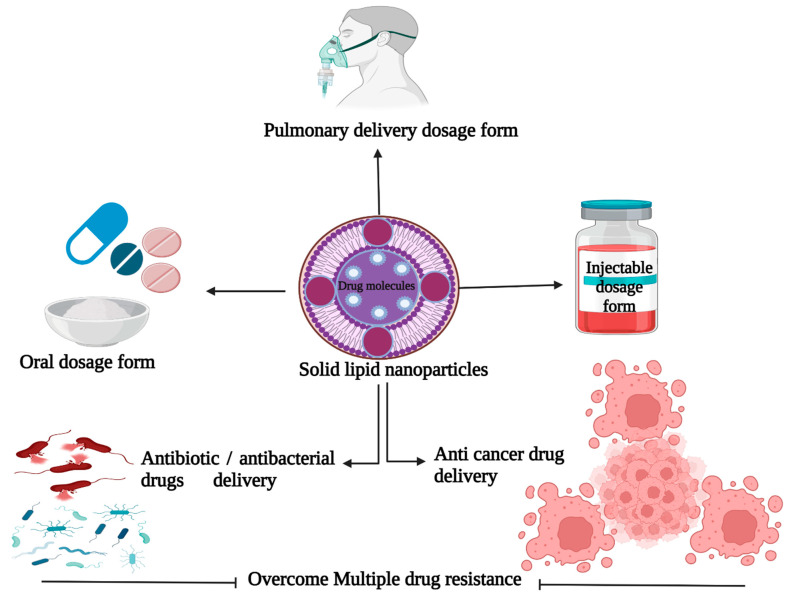
Schematic representation of solid lipid nanoparticles and their target specificities. (This figure was created with BioRender.com, Canada, accessed date: 3 October 2021).

Pharmaceutical firms have been using lipid materials that are solid at room temperature for several years to create a variety of formulations such as ointments, lotions, and suppositories [8]. Previous publications indicate that colloidal carriers such as liposomes, deformable liposomes, virosomes, ethosomes, and solid lipid nanoparticles are appropriate delivery systems due to their biodegradability and non-toxicity [9]. It is important to note that the sizes of SLNs are in the nanometer range and that their high affinity at the intracellular membrane level and in the intracellular space allows them to successfully permeate the cells [10]. The SLNs have gained a lot of attention because they can effectively deliver drugs and genes, and they can also be used for targeted therapies [11]. They have potential advantages over other nanoparticles due to their superior biocompatibility, increased drug loading capacity, and scalability. Lipids used in the manufacture of the matrix in SLNs are compatible with body immunity, and they have low toxicity levels and safe ratings. Basha and colleagues have posited that SLNs form dynamic, diverse, and modifiable drug delivery systems with increased potential for improving drug stability and controlling drug release from the matrix over time, and that packaging drugs in SLNs aids in resisting proteolytic degradation before the drugs reach the target sites [2]. Furthermore, pharma producers can use SLNs to create drugs for specific human organs based on their biochemical composition and type of reactions. In addition, the characteristics of the SLNs can be changed in order to improve their efficiency. This is particularly useful for drugs with low water solubility. Figure 1 shows a schematic depiction of SLNs and their target specificities in various perspectives. The present review focuses on the many types of solid lipid nanoparticles and traditional matrices utilized as drug delivery methods, in order to appreciate SLNs and their prospective applications in the pharmaceutical industry.

## 2. SLNs and NLCs for Drug Delivery

In terms of lipid-based therapeutic formulations, liposomes and various variants have been used since the 1960s, and they are considered the classic models. Previous reports indicated that liposomes are associated with inherent failures and poor properties with respect to polymer degradation, lack of large-scale production technology, fusion and drug leakage, degradation of the polymer matrix and cytotoxicity, phospholipid degradation, high production costs, and sterilization issues [12,13,14]. In 2002, Müller and colleagues proposed the terms solid lipid nanoparticles and nanostructured lipid carriers. These arose from a natural idea that combines the advantageous characteristics of NPs with those of non-toxic and biodegradable lipid components to form non-toxic and biodegradable parenteral emulsions that could be administered intravenously [15].

The nanocarriers are classified based on their compositions, route of administration, and target organs. There are two types of nanocarriers that can be distinguished based on their composition: solid lipid nanoparticles (SLNs) and nanostructured lipid carriers (NLCs). The SLNs have been promoted as viable alternatives to liposomes, emulsions, and polymeric nanoparticle carrier systems since 1990 [13,16]. They have a spherical morphology with an average size of 40 to 1000 nm, and they can be investigated using transmission electron microscopy and scanning electron microscopy.

## 3. Solid Lipid Nanoparticles

**Figure 2:**
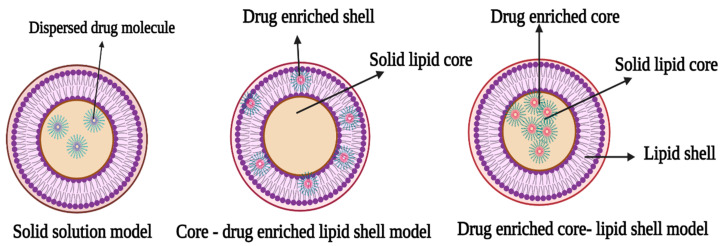
Schematic representation of types of solid lipid nanoparticles (The figure was created with BioRender.com, Canada, accessed date: 3 October 2021).

Solid lipid nanoparticles (SLNs) are spherical nanoparticles with a solid lipid core that contains the drug. The SLNs are innovative drug delivery vehicles that combine the benefits of polymeric nanoparticles with those of liposomes [15]. They were designed for sustenance of drug release profile, thereby reducing the need for recurrent drug administration, in addition to increasing the therapeutic effect of treatment and overcoming disadvantages inherent in traditional drug carriers [12,14]. The lipid components of a solid lipid nanoparticle matrix are in solid form at room and body temperatures; they are mostly super-purified waxes, triglycerides, and complex glyceride emulsions [3,12]. Different concentrations of surfactants that range from 0.5% to about 5% are included to ensure the stability of the drug carriers. Furthermore, the choice of the different components of the SLNs, especially the surfactant and lipid components, affects their quality and final physicochemical properties such as drug loading and particle size. In addition, SLNs may differ in properties owing to differences in formulations, preparation techniques, and production temperatures [9,15,16,17]. There are three models of drug entrapment. These models are represented in Figure 2 and described as follows:

### 3.1. Solid Solution Model

The solid solution model of SLNs is generated through cold homogenization, in which a solid drug and lipid solution are employed in the absence of a surfactant, and the drug molecules are spread throughout the matrix. They are ground in a homogeneous matrix during solidification of the solid drug components. Surfactants maintain the drug in the core, and drug availability is boosted by a combination of processes, including direct gastrointestinal absorption and the capacity to stimulate the lymphatic transport system.

### 3.2. Core-Shell Model (Drug-Enriched Shell)

In this model, the drug molecule is encapsulated in a core shell created by a thermal homogenization process to produce a shell rich in the drug component. The creation of a lipid core at the recrystallization temperature of cooling of the resultant dispersion results in the re-partitioning of the drug to the lipid phase in this technique [18].

### 3.3. Core-Shell Model (Drug Enriched Core)

The core-shell model with a drug-enriched core is made by dispersing drug molecules in melting lipid prior to cooling them, resulting in supersaturation of the drug dissolved in the lipid [19]. A membrane-like structure forms as the lipid recrystallizes around the drug-rich core.

According to an earlier publication, SLNs are superior to typical carriers because their liquid lipid complexity allows them to produce fewer defective crystals, thereby increasing their capacity to hold more medicinal components in their matrix. Furthermore, it has been suggested that the crystallinity and length of encapsulation of a drug are determined by their lipid nature; nano-formulation in bulky lipids has a complicated lattice arrangement that reduces drug leakage from the matrix [9,18]. Nanoparticle size, zeta potential, and polydispersity index (PDI) are all important properties of nanocarriers. It is important to remember that nanoparticle formulation takes into account thorough characterization in terms of size, shape, surface charge, and dispersion, for the sake of reproducibility in large-scale production. During manufacturing, there is a thorough sieving-and-mixing process that ensures that the final injectable nanoparticle system is homogenous and can easily penetrate through targeted cell membranes [13]. Other crucial factors include unspecific distribution, cellular internalization, drug efflux pumps, and interstitial fluid pressure, in addition to size, charge, and shape. Furthermore, after ingestion of drugs for different diseases, the nanoparticles are bound to plasma proteins, resulting in alterations in surface charges, affinities, and biological activities [19]. Positive charges on the surface of nanoparticles enhance their binding to cell membranes and normal tissues and promote platelet accumulation and other hemolytic processes.

## 4. Nanostructured Lipid Carriers

Nanostructured lipid carriers (NLCs) have emerged as a second generation of lipid nanoparticles in order to address the drawbacks of the first generation, i.e., SLNs. The NLCs are prepared using biodegradable and suitable lipids (solid and liquid) and emulsifiers. Incorporation of liquid lipids (oils) results in structural defects in solid lipids, leading to a less ordered crystalline arrangement that prevents drug leakage and provides a high drug load [20,21].

Nanostructured lipid carriers (NLCs) are created and marketed as superior drug carriers because, unlike SLNs, they are devoid of low drug-loading and ejection due to their synthesis from a liquid lipid and solid mixture with no clear crystalline structure. The NLCs have many significant properties such as higher loading capacity, reduced water content in the dispersion, and reduced or complete absence of evaporation of medication during storage. They are made up of a range of spatial lipids such as glycerides, and they feature unstructured crystals as well as extended distances between fatty acid chains in the glyceride components, thereby allowing for more drug accommodation [13]. Although NLCs possess significant properties, they have also been associated with cytotoxic effects, low stability, irritation, and sensitizing action of surfactants [22,23]. The NLCs are further classified into three sub-types: amorphous, multiple, and imperfect. The lipid composition of amorphous NLCs is unique. Most of them, for example, contain hydroxyl octacosanyl, isopropyl myristate, and hydroxyl stearate, which ensure that the solid liquid does not crystallize when cooled. The imperfect type is made by combining small amounts of oils with solid lipids so as to increase drug loading capacity. A study has shown that the multiple types of NLCs contain more lipids because drug solubility is higher in liquid lipids than in solid lipids; NLCs also have the advantage of modulating drug release in addition to superior drug loading and fewer drug expulsion issues [13].

## 5. SLNs as Antibiotic Delivery Agents

**Figure 3:**
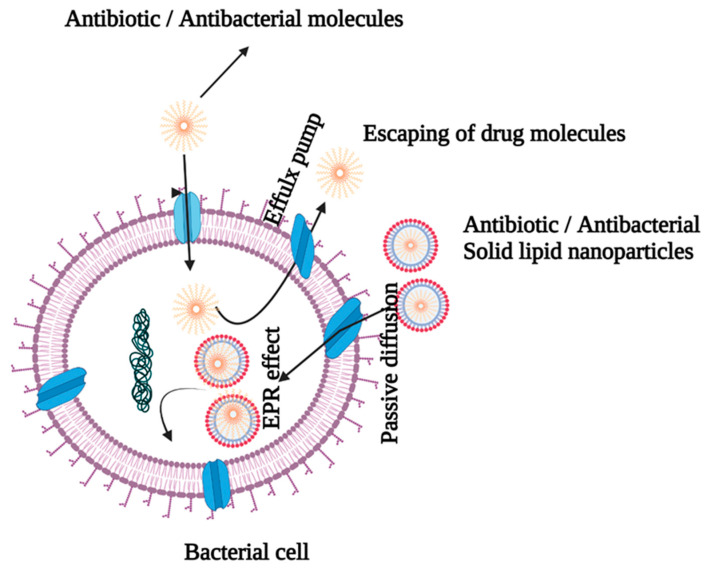
Antibiotic-entrapped SLN delivery by bypassing efflux pumps.

Bacterial resistance to a wide range of antibiotics and antibacterial treatments is a widespread occurrence that can be triggered by a variety of mechanisms, including efflux pumps and exocytosis [24,25]. Antibiotic resistance has paved way for the use of higher doses of antibiotics/antibacterials to combat infection, leading to increased toxicity and mortality [26]. Multiple antibiotic resistance in Gram-negative bacteria is caused by a decrease in outer membrane permeability and the creation of the R factor [24]. The SLNs are beneficial because they overcome drug resistance by providing a more effective delivery route for antibiotics/antibacterial agents, as shown in Figure 3.

The nature of the cell wall determines the interactions between SLNs and bacteria. It is critical that cationic SLNs be used to enable electrostatic interactions because the cell membranes of Gram-positive and Gram-negative bacteria are anionic in nature [26,27,28]. The SLNs are effective in reducing the risk of infection by bypassing first-pass metabolism and increasing intestinal lymphatic transport via the paracellular and transcellular channels of enterocytes, as well as endocytosis of phagocytic cells [29]. Lipids are appealing drug delivery vehicles because they are biocompatible with cell membranes. Moreover, lipids improve drug absorption via transcellular, paracellular, and lymphatic transport, thereby boosting bioavailability of drugs. There are a number of obstacles that must be overcome before the orally delivered antibiotics may be used effectively. A physical barrier is created in the GIT by the tight connection of the mucus layer that lines it, which impedes antibiotic absorption. Drug capture by the mucus layer, and the rapid turnover of mucus layer cells, limit drug absorption efficiency [30,31]. An earlier study indicated that doxycycline-encapsulated SLNs produced a superior effect against chronic brucellosis [32]. A study has revealed that SLNs could be potential delivery routes for increasing the antibacterial activity of rifampicin against *Brucella abortus* [33]. The ciprofloxacin solid lipid nanoparticle formula incorporating stearic acid (CIPSTE) had reduced particle size, good particle distribution, and a high percentage of drug encapsulation [14]. Another investigation suggested that pulmonary administration of amikacin SLNs enhanced the effect of the drug on cystic fibrosis-associated pulmonary infections since inhalation of the nanoparticles lowered dose frequency and improved the therapeutic index of the drug [34]. Moreover, the solid lipid nanoparticles (SLNs) of amikacin were designed for pulmonary delivery to reduce the dose or its administration intervals, leading to reduction in its toxicity, especially in long-term treatments [35]. The study also demonstrated that the SLN delivery system successfully delivered amikacin at lower doses via improved cellular penetration across the bacterial cell membrane [35].

## 6. SLNs as Delivery Vehicles for Anticancer Agents

**Figure 4:**
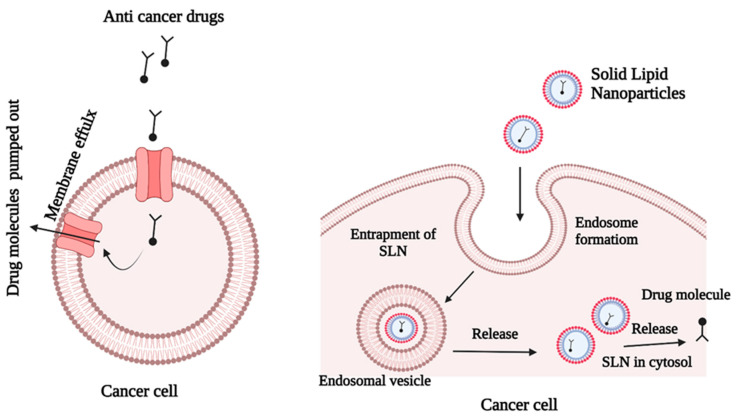
Escape mechanism of SLNs that contain anticancer drugs.

Cancer is characterized by uncontrolled cell division, resistance to cell death, and the capacity to metastasize to other tissues [36]. The most comprehensive cancer treatment is chemotherapy, which is given via traditional drug administration routes. However, this approach has several drawbacks, including low drug solubility, low specificity, high toxicity, and a low therapeutic index [37]. Cancer drug resistance is the most difficult obstacle to overcome in the treatment of cancer. Cancer cells frequently develop resistance to chemotherapy medications, resulting in treatment failure (Figure 4). It is possible that resistance to cancer medicines is caused by genetic alterations that result in corresponding changes in signaling pathways. Studies have shown that multi-drug therapy with diverse target mechanisms has higher treatment effectiveness than mono-drug therapy [24].

Extrinsic drug resistance and acquired drug resistance, which are the most common causes of chemotherapy failure, have significant negative impacts on the therapeutic outcomes of chemotherapy. Recent advances in nanotechnology have opened new options for treating tumors that have become resistant to conventional therapies [38]. Figure 4 shows that SLNs can overcome drug resistance by successful penetration into the cancer cells. The SLNs accommodate both hydrophilic and lipophilic anticancer agents, depending on the nanoparticle composition and preparation method. The drugs may be incorporated in three ways: (i) drug molecules may be dispersed homogeneously in the lipid matrix; (ii) drug molecules may be incorporated into the nanoparticles, and (iii) drug molecules may be incorporated into the shell of the nanoparticles. Docetaxel is a lipophilic natural compound with antimitotic activity and poor water solubility. These properties make docetaxel suitable for use in SLN formulations. A previous investigation revealed that SLN systems enhanced the production of cytotoxicity in MCF-7 breast tumor cells, when compared with the drug in its commercial form [39].

In vivo and in vitro studies have investigated the potential efficacies of assimilations of SLNs into other systems. The use of SLNs has been shown to have fewer negative effects, while simultaneously increasing the residence time and efficacy of cytotoxic medicines. Doxorubicin is a natural drug molecule with significant anticancer activity. Its administration produces numerous adverse effects, including heart issues. Doxorubicin can be incorporated into SLNs, and when combined with alpha-tocopherol succinate, which also has anticancer potential, it exhibited great cytotoxicity and absorption capacity in drug-resistant MCF-7 human breast and NCI ovarian cancer cells [40].

The chemotherapy treatment of solid tumors continues to pose serious challenges due to poor responses that have been predicted to be as low as 20% in esophageal cancer, pancreatic cancer, and ovarian cancer, despite the fact that solid tumors account for more than 80% of all cancers in humans [41]. An earlier study demonstrated that miRNA-200c was successfully entrapped in SLNs to avoid development of resistance. An investigation suggested that paclitaxel-loaded SLNs could be used to treat drug-resistant breast cancer cells. This was supported by findings in subsequent research. The activity of paclitaxel-SLNs against MCF-7 drug-resistant and drug-sensitive cells was shown to be substantial. A recent study reported that SLNs of doxorubicin produced promising potential as a useful therapeutic approach for overcoming the chemoresistance of adriamycin-resistant breast cancer [42].

## 7. SLNs as Delivery Vehicles for Antiviral Agents

Adefovir dipivoxil (ADV) is acyclic phosphonate nucleotide analogue of adenosine monophosphate with antiviral activity. The drug can be administered as oral medication for the treatment of hepatitis B virus infection (HBV) and herpes simplex virus infection (HSV) [43,44,45,46]. Adefovir dipivoxil-entrapped SLNs were developed by incorporating octadecylamine-fluorescein isothiocyanate and reported that SLN-ADV showed enhanced inhibitory effects in in vitro against hepatitis B surface antigen, hepatitis B antigen, and hepatitis B virus DNA levels [47]. In an animal model of herpes simplex virus infection, Kondel et al. (2019) established the sustained release of acyclovir from SLNs against the treatment of HSV-1 infection and concluded that acyclovir-SLNs can be offered as a single dosage [48]. Sorafenib is an FDA-approved anticancer drug used to treat hepatocellular carcinoma and advanced renal carcinoma. Previously published research established that sorafenib and superparamagnetic iron oxide nanoparticles (Sor-Mag-SLNs) are entrapped in solid lipid nanoparticles. Through sorafenib’s cytotoxic action, the Sor-Mag-SLNs limit cancer cell proliferation in human hepatocarcinoma HepG2 [49].

In 2016, a study found that acyclovir-SLNs had a greater encapsulation efficiency, a relatively higher loading capacity, and a preset good in vitro drug release profile [49]. Parthiban and Prakash (2020) stated that the SLN was successfully constructed as a sustained release carrier system for acyclovir, resulting in increased bioavailability, decreased toxicity, and higher antiviral effectiveness against HSV [50,51]. The antiretroviral protease inhibitor ritonavir is one of the drug-prescribed medications for HIV infections. An earlier study revealed enhanced encapsulation efficiency, improved antiviral effectiveness, and regulated release of ritonavir. Thus, in vitro antiviral experiments demonstrated that ritonavir SLNs can actively limit HIV virus development [52]. Favipiravir is an antiviral used to manage influenza, and that has the potential to target other viral infections [53]. Recently, favipiravir has been prescribed in the treatment of COVID-19. Recent finding suggests that favipiravir-SLNs showed antiviral activity against SARS-CoV-2 [54].

## 8. SLNs as Delivery Vehicles for Antifungal Agents

Drug distribution to the skin is well regarded as an effective method of treating a variety of dermatologic illnesses on a local level [55]. The development of novel ways for treating fungal infections on the skin requires the use of sophisticated carrier systems for a variety of drug molecules [56]. SLN formulations are being investigated as alternate delivery vehicles for ketoconazole oral dosing in the treatment of fungal infections [57]. An earlier study suggested that topical gels containing fluconazole-loaded SLNs have been created for the treatment of *Pityriasis versicolor* and have demonstrated a superior considerable rapid therapeutic index [58]. To overcome drug resistance in humans, a combinatorial drug delivery system that transports two or more medications to the targeted region in the human body, also known as a dual-drugs delivery system, provides a viable option [59]. *Candida albicans* is an emerging worldwide health threat that causes significant morbidity and mortality in immunocompromised and critical care unit patients [60,61]. SLNs loaded with clotrimazole (CLZ) and alphalipolic acid (ALA) are thought to be an effective treatment of *Candida albicans* mycosis [59]. Previously, it was hypothesized that miconazole-loaded SLN formulations for oral administration could be a novel strategy for boosting the antifungal efficacy of this medicine [62]. Fungal keratitis continues to be a serious sight-threatening condition. Ocular drugs are difficult to administer due to anatomical and physiological limitations. Recent research indicated that econazole-entrapped SLNs were successfully formulated, resulting in a potentially beneficial therapeutic approach for fungal keratitis [63].

## 9. Routes of Administration

The SLN technology has made significant advances in the treatment of a wide range of diseases. The encapsulation of lipophilic and hydrophilic drugs in SLNs reduces their degradation in the body, while also allowing for prolonged strategic drug release. Different types of nanoparticles have been designed for the following applications:

### 9.1. Oral Administration

The SNLs may be converted into conventional oral dose forms such as pellets, capsules, powders, and tablets. Moreover, SLN dispersion may be used in place of granulation fluid in a process such as wet granulation [19]. If the SLN dispersions are in powdered form, it is possible to tablet them directly by lyophilization and spray-drying after they have been prepared. In order to maximize cost-effectiveness, dried SLN powder may be packaged into gelatin capsules or produced as liquid polyethylene glycol 600 and packaged into soft gelatin capsules or converted into a dry powder and put into sachets after lyophilization [2]. Numerous studies have been conducted on bioactive compounds for SLNs for oral delivery. A study has successfully demonstrated that SLNs are suitable carrier systems for insulin administration via the oral route [64]. Furthermore, the solid matrix of SLN has been found to effective in protecting insulin from chemical breakdown in the gastrointestinal system, while simultaneously promoting insulin absorption through the intestinal wall. A recent study suggested that docetaxel-loaded solid lipid nanoparticles are promising drug carriers in the treatment of breast cancer and in prevention of metastasis [65]. In a different study, solid lipid nanoparticles (SLNs) coupled with transferrin in the treatment of breast cancer produced a substantial potential to transport tamoxifen citrate to the tumor cells, with improved therapeutic effects [66].

### 9.2. Parenteral Administration

The parenteral mode of administration is the most effective for delivering bioactive pharmacological compounds with narrow therapeutic index values and low bioavailability, particularly for medications recommended for unconscious patients [67]. Parenteral drug administration has benefited from significant technological advancements, leading to the creation of sophisticated systems that allow for drug targeting as well as sustained or controlled release of parenteral medications [68]. Proteins and peptide drugs are more prone to enzymatic degradation, a situation that necessitates frequent compensation when the drugs are taken orally. It has been reported that SLNs with controlled drug release mechanisms for parenteral administration are effective therapeutic strategies for avoiding increased patient adherence and frequent administration [69]. Parenteral administration of SLNs could take many forms, from intravenous to intraarticular. Several investigations on the pharmacokinetics and distribution of doxorubicin composition in SLNs in tissues have been conducted [2,37]. These studies showed that only the brain tissues of rabbits had doxorubicin. To improve distribution to the brain, stealth agents were added to the SLNs. Oral administration of doxorubicin, on the other hand, resulted in poorer bioavailability in the liver and heart.

### 9.3. Pulmonary Administration

Inhalation drug delivery is a non-invasive method of administration that offers many advantages for local therapy of airway diseases; it reaches the epithelium directly, bypasses first pass metabolism, and it has low toxicity [70]. A research study was carried out on pulmonary administration of amikacin-loaded SLNs, with the goal of boosting its concentration for the treatment of cystic fibrosis-associated lung infections in male rats [71]. Both free medication and cholesterol-loaded drug administration methods were used, including intravenous and pulmonary delivery routes. A previous study showed that pulmonary drug delivery produced fewer adverse effects in the kidney, with a longer drug dose interval than intravenous administration, resulting in better patient adherence [68,70]. Individuals who have difficulty adhering to SLN medications, or those who have kidney issues, may find that pulmonary administration of SLN medications advantageous. In addition to oral and parenteral administration, other major administration routes for SLN-packaged pharmaceuticals include ocular and rectal administration, each of which has its own set of advantages [67].

## 10. Miscellaneous

A study has shown that SLNs improved the pharmacokinetic parameters of the HIV protease inhibitor atazanavir [71]. A 2021 study discussed the importance of using lipid-based nanocarriers to deliver anti-retroviral drug molecules, as well as the specificity of the drug molecules delivered to the target [72]. A 2019 study reported the efficacy of acyclovir solid lipid nanoparticles that resulted in the sustained release of acyclovir achieved as a single dose [53]. Recently, a study reported that the non-mRNA peptide-based NVX-CoV2373 was associated with an adverse event similar to that of vaccine-induced myocarditis in a phase III clinical trial [73]. Interestingly, the SLN/DNA complex has been optimized as a lipoplex vaccine [74]. In a very recent study in 2022, it was reported that curcumin-entrapped SLNs in a microneedle transdermal patch were effective in the treatment of Parkinson’s disease [75].

## 11. Conclusions and Prospects

The SLNs offer enormous potential for developing treatments for several diseases, and they can be used as alternative colloidal drug delivery systems. Conventional medications pose toxicity risks to healthy cells. The current focus of research is on cell toxicity and inflammatory responses caused by SLNs, in order to develop models that would eliminate harmful side effects in their applications. Researchers are optimistic that the utilization of stealth SLNs and NLCs will be optimized and adjusted in the near future into forms such as nanoparticulate lipid carriers and targeted SLNs. These are expected to lessen side effects, while boosting the usefulness of the nanoparticles in anticancer treatments.

## Data Availability

Not applicable.
